# The Clinical and Economic Impact of Exacerbations of Chronic Obstructive Pulmonary Disease: A Cohort of Hospitalized Patients

**DOI:** 10.1371/journal.pone.0101228

**Published:** 2014-06-27

**Authors:** Francesco Blasi, Giancarlo Cesana, Sara Conti, Virginio Chiodini, Stefano Aliberti, Carla Fornari, Lorenzo Giovanni Mantovani

**Affiliations:** 1 Department of Pathophysiology and Transplantation, Università degli Studi di Milano, IRCCS Fondazione Ca’ Granda, Ospedale Maggiore Policlinico, Milan, Italy; 2 CESP, Research Centre on Public Health, Department of Statistics and Quantitative Methods, University of Milano – Bicocca, Monza, Italy; 3 Department of Health Sciences, University of Milano – Bicocca, Respiratory Unit, AO San Gerardo, Monza, Italy; 4 University of Napoli Federico II, Naples, Italy; University of Modena and Reggio Emilia, Italy

## Abstract

**Background:**

Chronic Obstructive Pulmonary Disease (COPD) is a common disease with significant health and economic consequences. This study assesses the burden of COPD in the general population, and the influence of exacerbations (E-COPD) on disease progression and costs.

**Methods:**

This is a secondary data analysis of healthcare administrative databases of the region of Lombardy, in northern Italy. The study included ≥ 40 year-old patients hospitalized for a severe E-COPD (index event) during 2006. Patients were classified in relation to the number and type of E-COPD experienced in a three-year pre-index period. Subjects were followed up until December 31^st^, 2009, collecting data on healthcare resource use and vital status.

**Results:**

15857 patients were enrolled –9911 males, mean age: 76 years (SD 10). Over a mean follow-up time of 2.4 years (1.36), 81% of patients had at least one E-COPD with an annual rate of 3.2 exacerbations per person-year and an all-cause mortality of 47%. A history of exacerbation influenced the occurrence of new E-COPD and mortality after discharge for an E-COPD. On average, the healthcare system spent 6725€ per year per person (95%CI 6590–6863). Occurrence and type of exacerbations drove the direct healthcare cost. Less than one quarter of patients presented claims for pulmonary function tests.

**Conclusions:**

COPD imposes a substantial burden on healthcare systems, mainly attributable to the type and occurrence of E-COPD, or in other words, to the exacerbator phenotypes. A more tailored approach to the management of COPD patients is required.

## Introduction

Chronic obstructive pulmonary disease (COPD) is a common disorder with significant health and economic consequences which will remain a challenge for the future. [1] It is a major cause of chronic morbidity and mortality worldwide and by 2030, is projected to be the third leading cause of death and rank seventh as burden of disease. [2], [3].

The natural course of COPD is affected by exacerbations (E-COPD), the definition and classification of which are a source of controversy, but for the purposes of health economics research, one of the earlier consensus definitions works well. Based on healthcare utilization, an E-COPD can be classified as follows: mild, when the patient experiences a worsening of respiratory symptoms which can be self-treated by increasing the usual medication; moderate, when the patient needs extra medication, namely systemic steroids and/or antibiotics; and severe, when the patient/caregiver is aware of an evident and/or rapid deterioration in condition, requiring hospitalization. [4] Exacerbations become more frequent and severe as COPD progresses, though it has recently been proved that exacerbation rates appear to reflect a susceptibility phenotype regardless of disease severity, the frequent exacerbator phenotype. [5], [6] E-COPD, particularly the severe forms, can have serious consequences on the patient’s quality of life, lung function, and life expectancy, as well as significantly increasing the burden in terms of health services and costs attributable to the disease. [7].

Most cost-of-illness analyses show that costs increase substantially with disease severity, and that a significant proportion of the economic burden of COPD can be attributed to exacerbations.[8]–[11] However, few studies have assessed the economic and social burden in relation to the type of E-COPD. [9] Moreover, data based on administrative healthcare databases about the frequency of exacerbations and the influence of multiple exacerbations on the clinical and economic burden of COPD are sparse in literature. [12], [13].

Our study provides an updated estimate of the COPD healthcare burden with regard to Italy and evaluates the existence of exacerbator phenotypes and its influence, both on the natural history of the disease and on healthcare cost in patients hospitalized for COPD in the general population.

## Materials and Methods

### Ethics Statement

We used administrative healthcare databases in the data warehouse (DWH) DENALI through a data sharing agreement with D.G. Sanità Regione Lombardia. Ethical consent was not required as this study was a secondary analysis of administrative anonymous information and data were analyzed anonymously.

### Study design and study population

This was a secondary analysis of the DWH DENALI, that organizes the various administrative databases of the publicly funded national healthcare system in Lombardy, a region in northern Italy numbering about ten million inhabitants. DENALI includes data regarding vital status, hospital discharges, pharmaceutical, and outpatient claims in the general population resident in Lombardy (Appendix S1– Section A).

Individuals ≥ 40 years old and covered by the regional health system of Lombardy who were hospitalized for a severe E-COPD from 1^st^ January to 31^st^ December 2006 were included. The first severe E-COPD registered during the one-year inclusion period was the index event and the day of admission to hospital was the date of entry in the study. Individuals were subsequently followed up until December 31^st^, 2009 or until death or migration outside Lombardy. Data regarding drug prescriptions, hospitalizations, and outpatient claims were extracted for each patient.

### Definition of E-COPD

E-COPD was defined as an event leading either to patients using antibiotics or corticosteroids or determining their hospitalization. The following algorithm was adopted to identify exacerbations in a three-year pre-index period and during follow-up.

A severe E-COPD was identified as hospitalization with a primary discharge diagnosis of COPD (international classification of diseases, ICD-9-CM, 491.2) or with a primary diagnosis of pneumonia (480–486), respiratory failure (518.8), pneumothorax (512.0, 512.8), heart failure (428), or severe pulmonary heart disease (415) in presence of a secondary diagnosis of COPD. Subsequent hospitalizations of the same patient were counted as one acute episode if they occurred within seven days after discharge. The E-COPD was considered as starting on the date of the first admission to hospital and ending on the discharge date of the last one. A moderate exacerbation was identified as prescription of respiratory antibiotics or corticosteroids used in the management of acute worsening of COPD symptoms (Appendix S1– Section B). Subsequent medications prescribed to the same individual accounted for one moderate E-COPD. Once moderate and severe exacerbations were separately defined, presence of a possible overlap between a moderate and a severe E-COPD was checked for. When treatment periods overlapped, two E-COPD were put together as one severe E-COPD, starting when the first one began and finishing when the second one ended. The same approach was used if less than seven days elapsed between the end of the previous E-COPD and the beginning of the following one.

### Baseline measurement and study groups

Comorbidities and Charlson index were computed at index event, based on the discharge diagnoses (ICD-9-CM) reported in all the hospitalizations registered in the pre-index period and in the index event. [14], [15] The study population was classified according to the number and type of E-COPD occurring over a three-year period before the index event. Three groups of patients were identified: those who had at least one severe E-COPD before the index event (Group A), those who had only moderate E-COPD (Group B) and those who did not have any E-COPD (Group C).

### Outcomes

The primary outcomes analysed during follow-up were all-cause mortality extracted from vital status records and E-COPD. Mortality at index event was defined as death occurring for any reason either during the relative hospitalization or within seven days after hospital discharge.

### Healthcare costs and resources use

Healthcare costs (€) in the follow-up were analysed from the perspective of the Italian National Health Service. Costs were quantified using charges, i.e. the amount of money the health system reimbursed to providers of care. Healthcare resources were divided into three main cost categories: hospitalizations, prescriptions of drugs and outpatient claims. For each category, the cost of resources connected to the management of COPD was highlighted. Hospital discharges were divided into hospitalizations for E-COPD and for other reasons. Prescriptions were divided into: antibiotics or corticosteroids used in the treatment of exacerbations, drugs for obstructive airway diseases, cardiovascular, and other treatments. Outpatient use of resources was split into three categories: diagnostic tests usually performed in COPD patients (pulmonary function tests, respiratory pattern examinations, 6-minute walking test, chest computed tomography or radiography, C-reactive protein and sputum examination), all the other diagnostic tests, and check-ups and general visits. A further detailed analysis was performed on pulmonary function tests, including: spirometry and lung volumes, body plethysmography, diffusion capacity, bronchial provocation, and reversibility testing.

### Statistical analysis

The statistical significance of differences among groups was tested adjusting for age and gender with the Cochran-Mantel-Haenszel test for categorical variables, ANOVA for continuous variables and Poisson regression for counting processes. The Kruskal-Wallis test was used to test differences in the mean follow-up time. The chosen significance level was 0,05 and Bonferroni correction was used for multiple comparisons.

The survival and the probability of having an E-COPD during the follow-up period were described using Kaplan Meier curves. Difference among groups was tested using Log-rank and the Wilcoxon test. We computed the rate of total severe plus moderate E-COPD, and of severe and moderate E-COPD taken separately, during follow-up, adjusting for age and gender. Rates are expressed per one person-year and confidence intervals (CI) were computed using Normal approximation. The impact of number and type of exacerbations experienced on mortality risk was analysed using a Cox model with death as the dependent variable, the number of severe and moderate E-COPDs as two time-dependent covariates, and age, gender, co-morbidities (Charlson index) and history of E-COPD as fixed covariates.

Cost of healthcare resources is reported as annual mean per person. In the estimation of the annual means we needed to account for right censoring, due to the fact that several patients left the cohort before the follow-up period was completed. Therefore, the Bang and Tsiatis partitioned estimator was used. [16] Confidence intervals for the estimates were calculated using the bootstrap method with 100 iterations.

## Results

### Study population

A total of 15857 patients –9911 males; mean (SD) age: 76 (10) years - who were hospitalized for an E-COPD (index event) during 2006 was identified. Among those, 36% belonged to group A, 53% to group B and 11% to group C (Table 1). At index event, the mean age did not vary in relation to history of E-COPD. Prevalence of men was lower in absence of history of E-COPD (group C) and it increased in case of history of severe E-COPD (Group A) versus only moderate E-COPD. Patients without E-COPD in the pre-index period (group C) had better clinical conditions: the Charlson index mean, as well as the prevalence of some of the comorbidities, were significantly lower than in groups A and B.

**Table 1:** Main characteristics of the population at index event in relation to the three study groups.

	Study population	Group A	Group B	Group C
N (%')	15857	5625 (36)	8436 (53)	1796 (11)
N. of Male (%)	9911 (63)	3780 (67)	5130 (61)^1^	1001 (56)^12^
Age - mean (SD)	76 (9.66)	75 (8.74)	76 (9.90)	76 (11.13)
Comorbidities'', N (%)				
Congestive Heart Failure	4889 (31)	1903 (34)	2474 (30)^1^	512 (29)^1^
Diabetes	3068 (17)	1230 (22)	1545 (18)^1^	293 (16)^1^
Cerebrovascular disease	2614 (17)	918 (16)	1384 (16)	312 (17)
Renal Disease	1722 (11)	672 (12)	906 (11)^1^	144 (8)^12^
Any malignancy	1959 (11)	620 (11)	930 (11)	109 (6)^12^
Peripheral Vascular Disease	1405 (9)	580 (10)	714 (9)^1^	111 (6)^12^
Myocardial Infarction	1253 (8)	456 (8)	674 (8)	123 (7)
Mild Liver Disease	115 (7)	469 (8)	537 (6)^1^	109 (6)^1^
Dementia	629 (4)	177 (3)	350 (4)	102 (6)^12^
Charlson Index'' - mean (SD)	1.6 (1.97)	1.7 (1.93)	1.7 (2.06)	1.3 (1.62)^ 12^

Group A: patients with at least one severe exacerbation of chronic obstructive pulmonary disease (E-COPD)in the three-year pre-index period; Group B: patients with only moderate E-COPD in the three-year pre-index period; Group C patients without E-COPD in the three-year pre-index period.SD = standard deviation.

' % of the whole population.

'' COPD is not considered as a comorbidity.

1 p<0.05 vs group A, ^2^ p<0.05 vs group B.

### Study outcomes

**Figure 1:**
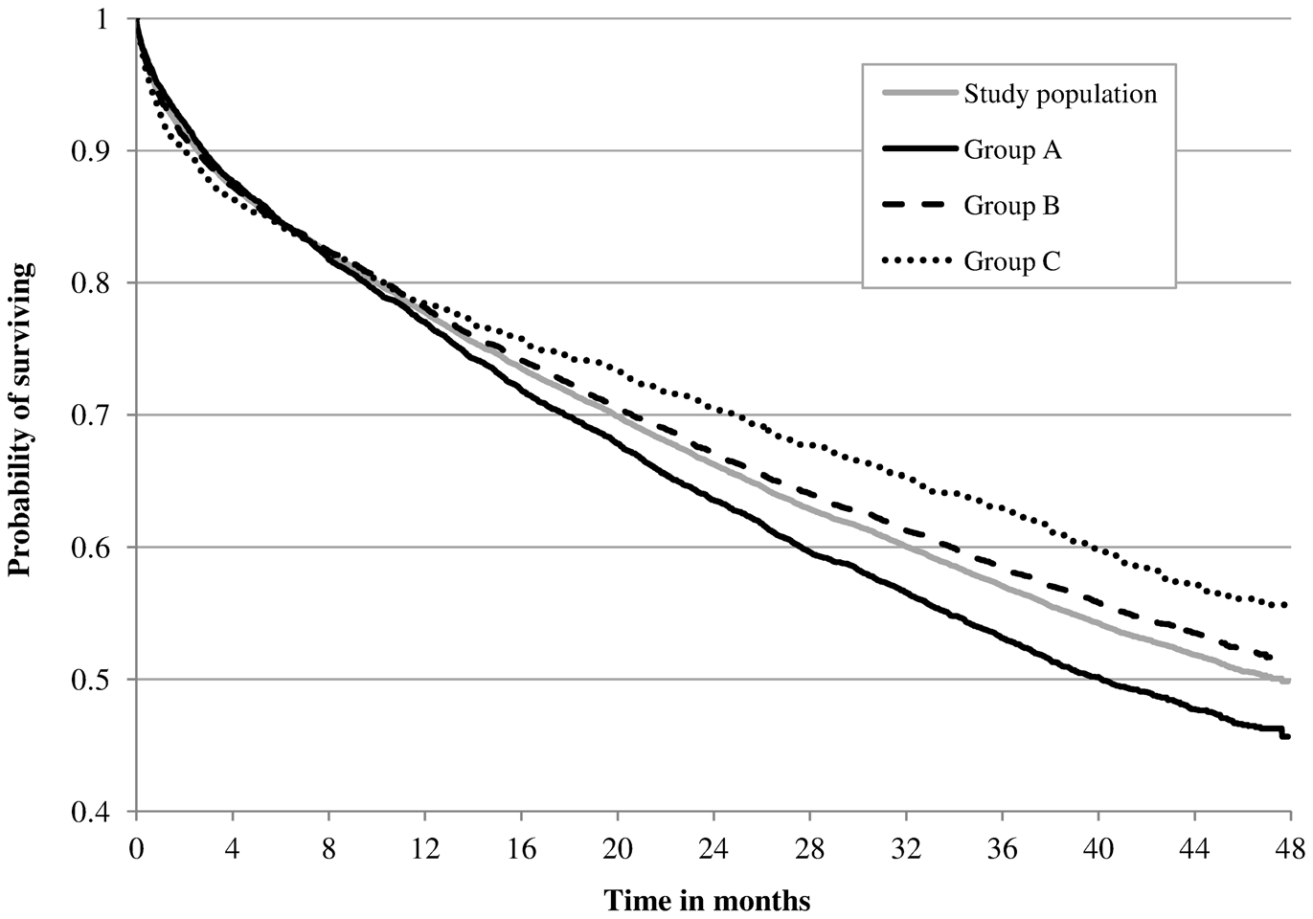
Probability of survival during follow-up in relation to the three study groups. Group A: patients with at least one severe exacerbation of chronic obstructive pulmonary disease (E-COPD) in the three-year pre-index period; Group B: patients with only moderate E-COPD in the three-year pre-index period; Group C patients without E-COPD in the three-year pre-index period. Differences between groups are significant (p<0.05, Log-rank and Wilcoxon tests).

During a mean (SD) follow-up time of 29 (16) months, 47% of subjects died with a higher percentage of deaths in presence of history of E-COPD and of severe type (Table 2). At index event mortality was 5% with no differences among groups. Individuals with a history of severe E-COPD had the lowest probability of surviving and patients without a history of exacerbation had the highest one (Figure 1).

**Table 2:** Main outcomes of the study population, in relation to the three study groups.

	Study population	Group A	Group B	Group C
Follow-up time (months) – mean (SD)	29 (16.31)	29 (16·41)	29 (16.24)	30 (16.29)
Death at index event, N(%)	760 (5)	253 (5)	403 (5)	104(6)
Death, N(%)	7452 (47)	2887 (51)	3826 (45)^1^	739 (41)^12^
Total E-COPD				
N. of subjects with at least one, N (%)	12856 (81)	4867 (87)	6871 (82)^1^	1118 (62)^12^
Annual rate' (CI 95%)	3.2 (3.15–3.20)	4.3 (4.19–4.31)	2.9 (2.88–2.94)	1.2 (1.13–1.21)
Severe E-COPD				
N. of subjects with at least one, N (%)	6878 (43)	3648 (65)	2823 (34)^1^	407 (23)^12^
Annual rate' (CI 95%)	0.5 (0.51–0.53)	0.9 (0.91–0.97)	0.3 (0.30–0.32)	0.2 (0.17–0.20)
Moderate E-COPD				
N. of subjects with at least one, N (%)	12045 (76)	4459 (79)	6585 (78)	1001 (56) ^12^
Annual rate' (CI 95%)	2.7 (2.64–2.68)	3.3 (3.26–3.36)	2.6 (2.57–2.63)	1.0 (0.95–1.03)

Group A: patients with at least one severe exacerbation of chronic obstructive pulmonary disease (E-COPD) in the three-year pre-index period; Group B: patients with only moderate E-COPD in the three-year pre-index period; Group C patients without E-COPD in the three-year pre-index period. The index event is not included. SD = standard deviation.

'Age and sex adjusted rate per one person-year.

1 p<0.05 vs group A, ^2^ p<0.05 vs group B.

**Figure 2:**
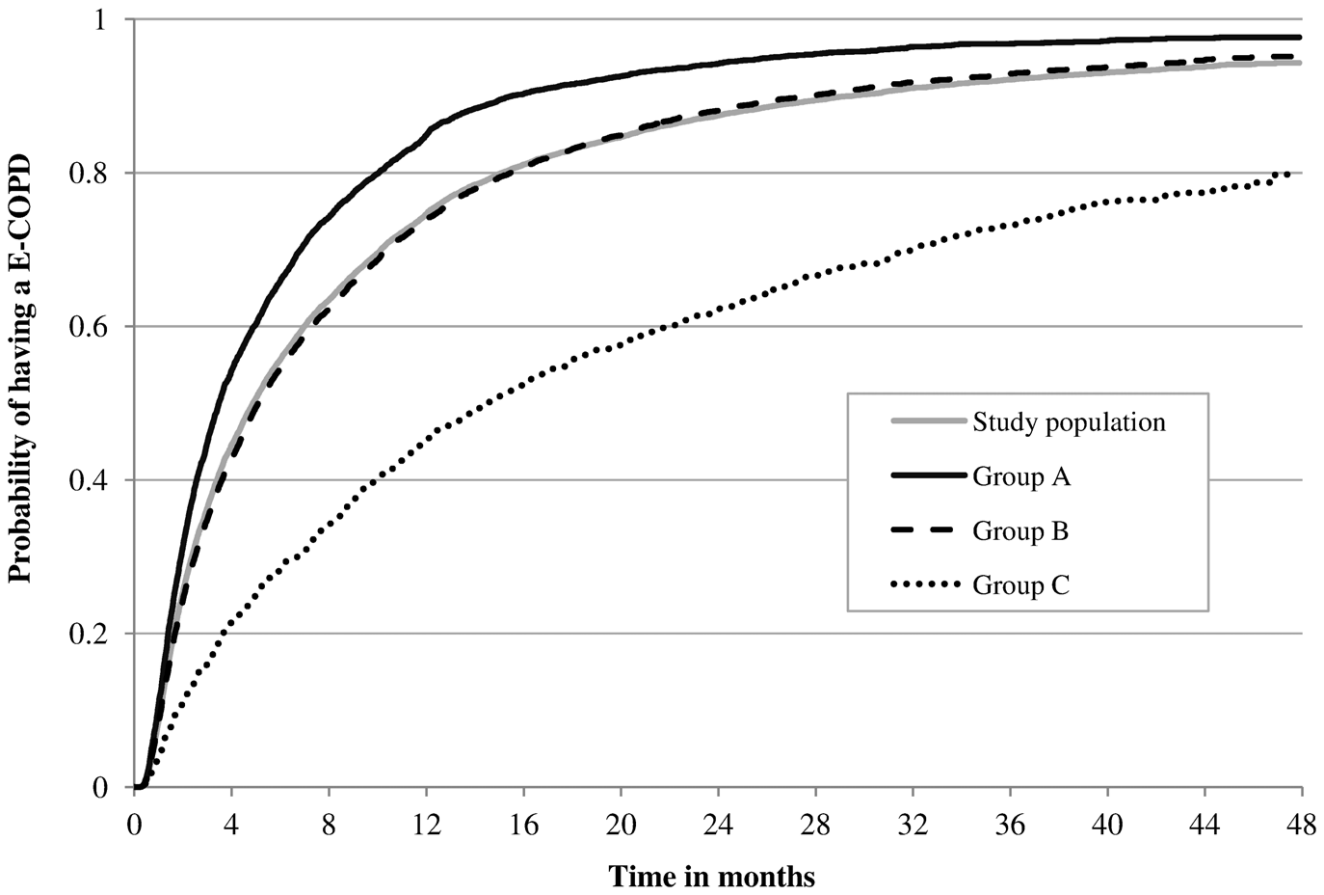
Probability of having an E-COPD during follow-up in relation to the three study groups. Group A: patients with at least one severe exacerbation of chronic obstructive pulmonary disease (E-COPD) in the three-year pre-index period; Group B: patients with only moderate E-COPD in the three-year pre-index period; Group C patients without E-COPD in the three-year pre-index period. Differences between groups are significant (p<0.05, Log-rank and Wilcoxon tests).

Patients in group A were more likely to experience a new E-COPD in the follow-up, in particular a severe one, than patients in groups B and C (Table 2). Moreover, patients in groups C were least likely to have moderate E- COPD. The probability of having a new E-COPD was 75% at one year from index event in the entire population, it increased to 92% at three years (Figure 2). The probability of experiencing a new E-COPD was higher in presence of a history of E-COPD at any time of follow-up: it was significantly highest in group A, then groups B and C followed in decreasing sequence. After the index event a patient had on average three E-COPD per year, of which less than one were severe and the remainders were moderate(Table 2). Patients in group A had the highest rate of both moderate and severe E-COPD and group B had higher rates than group C.

**Figure 3:**
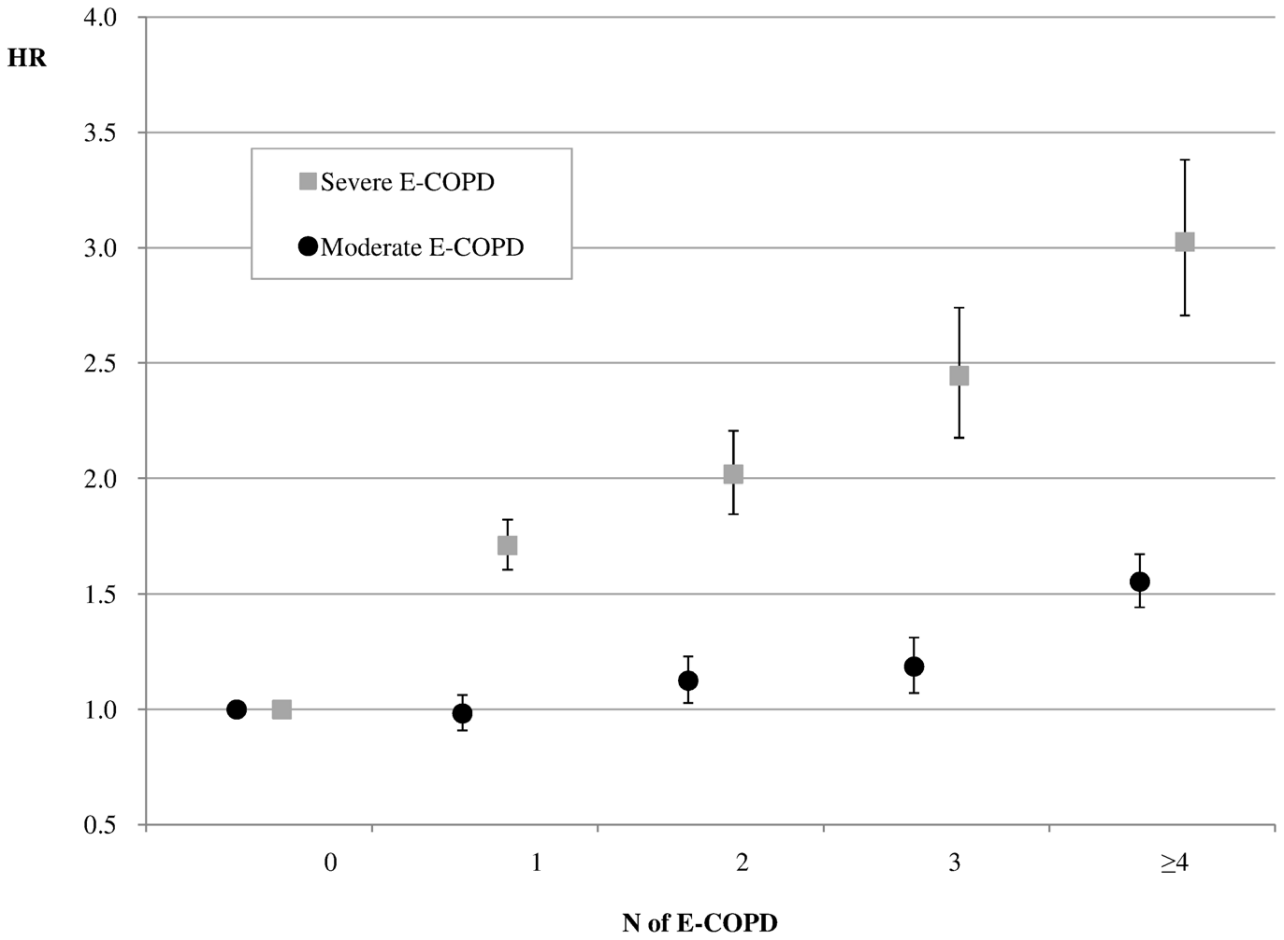
Hazard Ratio of death (HR, 95%CI) by number (N) and type of E-COPD experienced during follow-up. Time-dependent Cox model adjusted by age, sex, comorbidities (Charlson index) and history of E-COPD in the three-year pre-index period.

The effect of E-COPD, in terms of number and type, on the mortality risk is depicted in Figure 3. Mortality risk significantly raised in relation to increasing number of E-COPD experienced during the follow-up period. Moreover, the severity of the E- COPD significantly affected mortality risk.

### Cost and use of healthcare resources

The mean hospital cost for the index event in 2006 was 3985€ (95%CI 3913–4054) and it was highest in group A. The mean (95%CI) annual healthcare cost per patient in the follow-up was 6725€ (6590–6863), of which 71% was due to hospitalizations, 19% to prescriptions and 10% to outpatient visits and exams (Table 3). The direct medical cost related to COPD management amounted to 34% of the total cost. In detail the health system spent yearly 1705€ (1653–1764) per patient for hospitalized E-COPD, 48€ (47–49) for antibiotics or corticosteroids specific for the treatment of E-COPD, 496€ (487–507) for drugs for obstructive airway disease and 38€ (37–39) for outpatient diagnostics. In the breakdown of the total expense related to COPD treatment (hospitalization with a diagnosis of COPD, drugs for E-COPD, respiratory drugs and diagnostics for COPD, hospitalizations continued to account for more than 70%, the portion related to pharmaceutical increased to 24%, while outpatient diagnostics accounted for only 2%.

**Table 3:** Mean annual cost (€) per person of healthcare resources with 95%CI of the study population.

	Study population	Group A	Group B	Group C
Total Healthcare Resources	6725 (6590–6863)	8779 (8547–9005)	5999 (5839–6176)	3968 (3697–4283)
Hospitalizations	4753 (4645–4859)	6658 (6458–6871)	3957 (3819–4095)	2757 (2553–2979)
Diagnosis of E-COPD	1705 (1653–1764)	3268 (3139–3384)	945 (888–1000)	564 (502–651)
Other Diagnoses	3048 (2982–3126)	3390 (3254–3532)	3012 (2905–3117)	2193 (2039–2376)
Prescriptions	1269 (1245–1295)	1469 (1435–1506)	1253 (1212–1289)	756 (707–804)
Drugs for E-COPD’	48 (47–49)	63 (61–65)	45 (44–47)	16 (15–17)
Respiratory Drugs”	496 (487–507)	669 (648–686)	438 (428–452)	253 (229–275)
Cardiovascular Drugs+	234 (225–243)	218 (205–231)	252 (236–266)	201 (189–215)
Other Drugs	437 (419–453)	434 (413–455)	478 (450–508)	260 (231–292)
Outpatient Visits & Exams	703 (630–784)	652 (551–780)	789 (662–908)	455 (342–600)
Diagnostics for COPD∼	38 (37–39)	41 (39–44)	39 (37–40)	24 (22–27)
Other Diagnostics	619 (547–700)	559 (455–686)	705 (578–824)	400 (288–544)
General Visits	46 (45–46)	52 (50–53)	45 (44–46)	31 (29–33)

Group A: patients with at least one severe exacerbation of chronic obstructive pulmonary disease (E-COPD) in the three-year pre-index period; Group B: patients with only moderate E-COPD in the three-year pre-index period; Group C patients without E-COPD in the three-year pre-index period. The index event is not included.

'Drugs used to treat COPD exacerbation. ''Drugs for obstructive airway diseases - Anatomical therapeutic Chemical (ATC) Classification R03 -. ^+^Drugs for the cardiovascular system - ATC C -. ∼Pulmonary function tests, respiratory pattern examination, 6-minute walking test, chest computed tomography or radiography, C-reactive protein and sputum examination.

Patients in group A showed the significantly highest total healthcare cost per patient and patients in group B had higher per patient cost than patients in group C. Differences between groups A and B in the per patient cost were mainly attributable to differences in the expense due to hospitalizations for E-COPD and prescriptions of drugs related to COPD treatment, as well as diagnostics for COPD and general visits, while differences between group C and the other two groups were detected in all cost categories.

Up to 75% of the study population didn’t present any claim for pulmonary function tests at the end of follow-up, with differences among groups: the percentage of patients without exam was highest in group C and it was lowest in group B (Table 4). The mean annual cost per person of pulmonary function testing was 11€ (10–11) with a lower cost in group C. The cost didn’t change by year of follow-up and neither did the percentage of patients with at least one exam each year, which was about 15% with even lower values in group C.

**Table 4:** Mean annual cost (€) per person with 95%CI and resource consumption of pulmonary function test.

	Study population	Group A	Group B	Group C
N. of subjects with exam in (%)				
first year	2412(15)	829 (15)	1391 (17)^1^	192 (12)^12^
second year	1822 (15)	683 (16)	1010 (15)	129 (11)^12^
third year	1497 (14)	556 (16)	835 (14)	106 (9)^12^
entire follow-up	3904 (25)	1386 (25)	2215 (26)^1^	303 (17)^12^
Cost - annual mean (95% CI)	11 (10–11)	12 (11–13)	11 (10–12)	6 (5–7)

It includes spirometry and lung volumes, body plethysmography, diffusion capacity, bronchial provocation and reversibility testing.

Group A: patients with at least one severe exacerbation of chronic obstructive pulmonary disease (E-COPD) in the three-year pre-index period; Group B: patients with only moderate E-COPD in the three-year pre-index period; Group C patients without E-COPD in the three-year pre-index period.^1^ p<0.05 vs group A, ^2^ p<0.05 vs group B.

## Discussion

The present study evaluates the healthcare burden associated with COPD patients followed for up to three years after discharge for an episode of E-COPD. It also examines the presence of subgroups defined by exacerbation phenotypes and their influence on the progression of disease and healthcare costs. The main findings of the study are: 1) over a mean follow-up time of 2.4 years, 81% of patients had at least one moderate or severe E-COPD with an annual exacerbation rate of 3.2 per person-year and an all-cause mortality rate of 47%; 2) on average, the health system spent 6725 € per person per year, 34% of which could be attributed to COPD management; pulmonary function test was still underused; 3) the type and history of E-COPD impacted the occurrence of new E-COPD and mortality as well as healthcare use and costs during follow-up.

Our data report an high exacerbation rate over a long follow-up, with an annual rate of 3.2 per one person-year, and of 0.2 for severe exacerbations. Various authors have estimated the annual E-COPD rate from a low of 0.5 to a high of 3.5 per patient, but 3.0 per year represents the upper limit reported in literature in those studies with long-term follow-up. [17], [18] This high rate may also be influenced by the population characteristics, all patients had at least one hospitalisation for E-COPD. Moreover, we confirm the existence of exacerbator phenotypes and the importance of exacerbation history (number and type) in order to define them. [6], [12] Patients with a history of severe E-COPD are most likely to experience a new E-COPD with the highest annual exacerbation rate while patients with no history of E-COPD are least likely to experience new exacerbations with the minor exacerbation rate. These findings suggest that patients may not return to the previous stable state after an exacerbation, but there may be increased airway inflammation, lung function decline, increased bacterial colonization and thus increased susceptibility to further exacerbations. [19].

In our population, the probability of death was in line with mortality figures reported by other studies. [20]–[22] History of exacerbations was associated with a higher probability of death during the follow-up but not with mortality at index event, confirming a higher risk in relation to exacerbation frequency. [20] We also found that severe exacerbation history increases mortality risk. Moreover we found a positive relationship among mortality and both severity and number of E-COPD experienced during the follow-up at same history of E-COPD and comorbidities. The impact of occurrence and type of exacerbations on mortality can be explained by the level of both local and systemic inflammation characterizing an E-COPD that can lead to the development of adverse outcomes, including cardiovascular events. [23], [24].

The mean annual total healthcare cost was 6725€ per patient, 34% of which could be attributed to COPD management. In line with previous studies, the major driver of the direct medical cost in COPD patients was attributable to hospitalizations. [9], [10] Moreover, we confirm that pulmonary functional test was still underused, although the relative expense is low. [25], [26] On the other hand, performing spirometry may not be associated with significant improvement in the management of COPD. [27] Further studies are thus needed to investigate more closely the role of spirometry in the follow-up of COPD patients.

The type and frequency of exacerbations affect direct healthcare costs for society. In our population, patients with history of E-COPD accounted for a higher mean annual per patient cost than patients without any history. Moreover, patients with a history of severe exacerbations had the highest cost. A history of E-COPD therefore suggests a future frequent exacerbator phenotype, which leads to higher healthcare costs and use, as recent US studies have reported. [10], [12], [28] Furthermore, the healthcare costs increased in proportion to the severity of E-COPD, since a severe exacerbation requires hospitalization, at an average cost in our study of 3985 €, which is consistent with previous data. [29].

The present study has some limitations, mainly inherent to the use of healthcare administrative data. Although our definition of E-COPD fails to capture mild exacerbations and may have included some hospitalization that do not strictly refer to severe E-COPD (current guidelines definition), it is a recognized classification in healthcare economics research. Moreover, the population in study may underrepresent infrequent exacerbators and no clinical and functional data were available regarding either severity of COPD or causes of death. Finally, in our economic analysis, there were no data available as regards indirect costs, or costs related to emergency call-outs and general practitioner visits. Since general practitioners are paid on a capitation basis, the cost does not change in relation to the illness or the number of visits, so it is not relevant to economic evaluations. Indirect cost is difficult to estimate, and few studies have attempted to do so, although it is well-known that COPD affects quality of life and consequently leads to production losses, generating a substantial burden. [5] However, the use of administrative databases does provide a range of data not necessarily available from other sources with such low budget efforts. Our study is strengthened by the evaluation of the COPD burden in a large population outside strict selection criteria and by a potential follow-up time of at least three-years, a duration widely accepted as being long enough to capture significant endpoints.

## Conclusions

This study provides updated estimates of direct healthcare costs in COPD patients beyond their individual characteristics and the time duration of clinical trials. Secondly, it gives more strength to the concept of t exacerbator phenotypes, since both the type and frequency of exacerbations have a substantial impact on the patient’s quality of life and healthcare resource use and costs. The main implication of the study is the importance of prevention of exacerbations as the principal means of providing both clinical benefit and reducing healthcare cost in COPD patients. A flood of data shows that pharmacological interventions and vaccinations are able to significantly improve clinical outcomes. [30], [31] An improvement in COPD management with appropriate therapies, as well as correct prophylactic measures, would also clearly be cost-effective for healthcare systems.

## Supporting Information

Appendix S1 **Supporting information on methods.** The file contains two sections. Section A gives more details on the structure and content of the data warehouse DENALI. Section B describes the criteria used to define a moderate exacerbation of COPD.
(DOCX)
