# Supplementary material for: The Clinical and Economic Impact of Exacerbations of Chronic Obstructive Pulmonary Disease: A Cohort of Hospitalized Patients
Source: PLoS One. 2014 Jun 27;9(6):e101228. doi: 10.1371/journal.pone.0101228 (PMC4074190; doi:10.1371/journal.pone.0101228)
Supplement: Appendix S1 — Supporting information on methods. The file contains two sections. Section A gives more details on the structure and content of the data warehouse DENALI. Section B describes the criteria used to define a moderate exacerbation of COPD. (DOCX) [file pone.0101228.s001.docx]

**Section A. The data warehouse DENALI**

Healthcare administrative databases related to several services provided by the Italian Healthcare Service (HS) are created at regional level for administrative purposes. The administration of Lombardy, a region of the North of Italy, allowed the creation of a data warehouse (DWH) where its administrative healthcare databases are organized and available for scientific medical research. Multiple heterogeneous archives were joined together in a single storage using each patient as an indicator. Both data storage and software to create the DWH were named DENALI. This software integrates data into a semantically consistent store according to a data model suited to support structured and ad hoc queries. Because of this, DENALI data are cleaned, transformed and evenly catalogued. Furthermore, one of the distinguishing features of this software is the probabilistic reconstruction of links among databases without a sole identifier and which could have missing, defective or incorrect records.^1, 2^

DENALI includes data related to vital status, hospital discharges (HDs), pharmaceutical and outpatient claims related to the general population, as Italy has a single-payer national healthcare system. Historical vital records are registered for all residents in Lombardy and for all people treated in the healthcare facilities of this area. The database of HDs contains all the hospitalizations in both public and private hospitals funded by the HS. Pharmaceutical claims refer to prescription of drugs delivered at pharmacies located in Lombardy. Outpatient claims register specialists’ visits, diagnostics and medical treatments performed at healthcare facilities located in Lombardy. General practitioner (GP)’s visits are not included in Italian healthcare administrative databases because GPs are paid on a capitation basis. Moreover rehabilitation resources are not registered in the administrative databases that compose the DWH.

**Section B. Definition of Moderate Exacerbation of COPD**

Moderate exacerbation of COPD (E-COPD) was identified by the prescription of respiratory antibiotics or corticosteroids reported in the following table. Prescriptions of either corticosteroids more than 24 days, or clarithromycin more than 28 days or other antibiotics more than 16 days were considered as chronic treatments and did not define a moderate exacerbation. The expected duration of therapy for each prescription was calculated based on daily doses reported in the patient information leaflets.

When a prescription was registered during the time period of coverage of a previous one or during the seven days after the end of the period, both prescriptions accounted for one moderate exacerbation. When drugs prescribed during one exacerbation showed the same constituent, the relative durations of therapy were cumulated; otherwise, the longest time period was kept to determine dates of starting and ending of the exacerbation episode.

Table. Drugs selected to identify moderate exacerbations

| **Corticosteroids (ATC- Anatomical Therapeutic Chemical Classification System)** |
| --- |
| Bentalan (H02AB01), Betametasone FMM (H02AB01), Capital (H02AB02), Celestone (H02AB01), Cortiron (H02AA03), CortoneAcetato (H02AB10), Decadron (H02AB02), DecadronFosfato (H02AB02), Deflan (H02AB13), Deflazacort FG (H02AB13), Deltacortene (H02AB07), Deltacortenesol (H02AB06), Depo Medrol (H02AB04), Dirahist (H02BX), Flantadin (H02AB13), Flebocortid (H02AB09), Kenacort A (H02AB08), Ledercort (H02AB08), Medrol (H02AB04), Megacort (H02AB02), Soldesam (H02AB02), SoluCortef (H02AB09), Solu Medrol (H02AB04), Triamvirgi (H02AB08), UltralanOrale (H02AB03), Urbason (H02AB04). |
| **Antibiotics (ATC )** |
| Beta-lactam antibacterials, penicillins (J01C), Combinations of sulfonamides and trimethoprim (J01EE01), Clarithromycin (J01FA09), Ciprofloxacin (J01MA02), Levofloxacin (J01MA12), Moxifloxacin (J01MA14). |

**References**

1 Fellegi IP, Sunter A. A theory of record linkage. *J Am Stat Ass* 1969;**64**:1183-210.

2 Newcombe HB. Handbook of record linkage, methods for health and statistical studies, administration and business. Oxford: *Oxford University Press*, 1988.
